# Crystal structure of 4-(2-meth­oxy­phen­yl)piper­azin-1-ium 3,5-dintrosalicylate

**DOI:** 10.1107/S2056989022006831

**Published:** 2022-07-12

**Authors:** Veerappan Subha, Thangaraj Seethalakshmi, Thangavelu Balakrishnan, M. Judith Percino, Perumal Venkatesan

**Affiliations:** a PG and Research Department of Physics, Government Arts College (Autonomous and Affiliated to Bharathidasan University, Tiruchirappalli), Thanthonimalai, Karur-639 005, Tamil Nadu, India; b Crystal Growth Laboratory, PG and Research Department of Physics, Periyar EVR Government College (Autonomous and Affiliated to Bharathidasan University, Tiruchirappalli), Tiruchirappalli-620 023, Tamil Nadu, India; c Unidad de Polímeros y Electrónica Orgánica, Instituto de Ciencias, Benemérita Universidad Autónoma de Puebla, Val3-Ecocampus Valsequillo, Independencia O2 Sur 50, San Pedro Zacachimalpa, 72960, Puebla, Mexico; d Department of Chemistry, Srimad Andavan Arts and Science College (Autonomous), Tiruchirappalli-620 005, Tamil Nadu, India; Universidade de Sâo Paulo, Brazil

**Keywords:** crystal structure, proton transfer salts, 1-(2-meth­oxy­phen­yl)piperazinium cation, 3,5-di­nitro­salicylic acid

## Abstract

The title salt [systematic name: 4-(2-methoxyphenyl)piperazin-1-ium 2-carboxy-4,6-dinitrophenolate], C_11_H_17_N_2_O^+^·C_7_H_3_N_2_O_7_^−^, exhibits secondary nitro­gen atoms (N—H) in the 2-meth­oxy­phenyl­piperazine (2MeOPP) cation, which is protonated with a phenolic hydrogen atom of 3,5-di­nitro­salicylic acid (DNSA). One of the oxygen atoms of the nitro group in the 3,5-di­nitro­salicylate anion is disordered over two orientations with occupancy factors of 0.65 (7) and 0.35 (7) . The 2-meth­­oxy­phenyl­piperazinium cation and 3,5-di­nitro­salicylate anion are linked in the asymmetric unit by a bifurcated N—H⋯O hydrogen bond, which formed is between the H atom in the protonated piperazinium unit of the cation and the carb­oxy­lic acid group in the anion. The piperazine ring adopts a chair conformation. The crystal structure features N—H⋯O and C—H⋯O hydrogen bonds inter­actions, which lead to the formation of a sandwich-like arrangement. Hirshfeld surface analysis was used to determine the relative contributions of various inter­molecular inter­actions, indicating that that H⋯O/O⋯H (38. 3%) and H⋯H (31. 8%) contacts are the major contributors.

## Abstract 2

The different intra and inter­molecular hydrogen-bonding inter­actions in the crystal structure of the title salt are discussed.

## 1. Chemical context

1-(2-Meth­oxy­phen­yl)piperazine is a substituted cyclo aliphatic amine with two nitro­gen atoms at opposite positions of the six-membered ring. A substituent 2-meth­oxy­phenyl group is attached to one of the nitro­gen atoms while the other has one attached hydrogen atom (*i.e.* the secondary nitro­gen atom, N—H). Piperazine and substituted piperazine derivatives are often used as inter­mediates for a wide range of pharmaceuticals, polymers, dyes, corrosion inhibitors and surfactants. In particular, (2-meth­oxy­phen­yl)piperazine derivatives are used as 5-HT_1A_ receptor ligands with reduced α1-adrenergic activity (Raghupathi *et al.*, 1991 ▸; Orjales *et al.*, 1995 ▸; Zhuang *et al.*, 1998 ▸). 1-(2-Meth­oxy­phen­yl)piperazine-impregnated filters have been used for the detection of iso­cyanates in air (Sennbro *et al.*, 2004 ▸). 1-Cinnamyl-4-(2-meth­oxy­phen­yl)piperazine derivatives are used as ligands for *D*_2_ and *D*_3_ dopamine and serotonin 5-HT_1α_ receptors (Penjišević *et al.*, 2007 ▸). The crystal structure of eleven protonated 4-(2-meth­oxy­phen­yl)piperazin-1-ium salts with eleven different substituted benzoic acids (namely, 4/2-chloro­benzoic acid, 4/2-bromo­benzoic acid, 4/2-iodo­benzoic acid, 2-fluoro­benzoic acid, 2-methyl­benzoic acid, 4-amino/4-nitro-benzoic acid, 3,5-di­nitro­benzoic acid and picric acid) and three aliphatic di­carb­oxy­lic acid [maleic acid, fumaric acid and (2*R*,3*R*)-tartaric acid] salts and their supra­molecular features have been reported (Harish Chinthal *et al.*, 2020 ▸). 
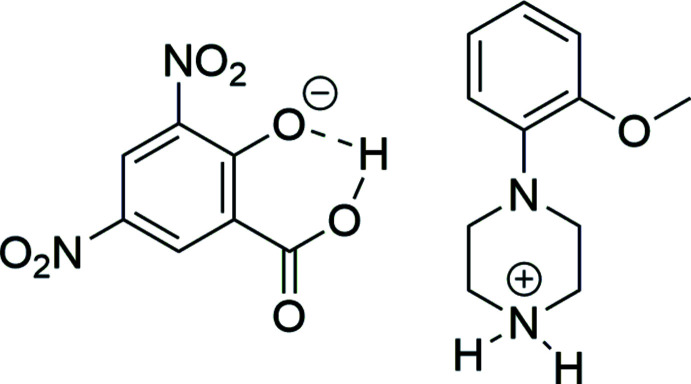


As a continuation of our earlier study on the crystal structure and supra­molecular analysis of a monohydrated 1:1 adduct of bis­(piperazine-1,4-diium), 3,5-di­nitro-2-oxidobenzoate and piperazine, we have now investigated the crystal structure of 1-(2-meth­oxy­phen­yl) piperazinium 3,5-dinitro­salicylate (I). In this study, the crystal structure, Hirshfeld surface (HS) analysis, structural features and various inter­molecular inter­actions that exist in the title protonated salt are reported.

## 2. Structural commentary

**Figure 1:**
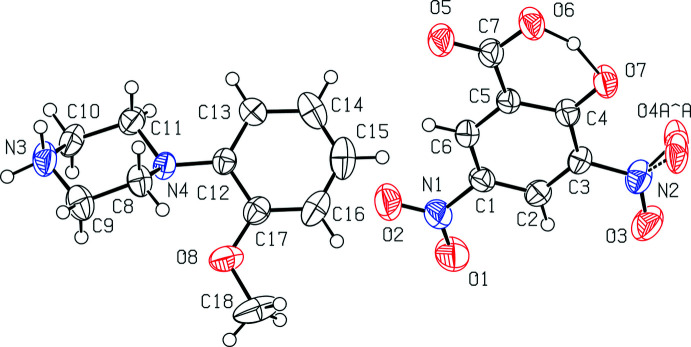
The mol­ecular structure of the title mol­ecular salt, (I), showing the atom-labelling scheme. Displacement ellipsoids are drawn at the 50% probability level.

The title salt crystallizes in the triclinic space group *P*ī with the asymmetric unit comprising one 2-meth­oxy­phenyl­piperazinium (2MeOPP)^1+^ cation and one 3,5-di­nitro­salicylate (DNSA)^1−^ anion (Fig. 1 ▸). The piperazine ring in the cation adopts a chair conformation with puckering parameters *Q* = 0.582 (3) Å, θ = 176.3 (3)°, φ = 338 (4)°. One of the oxygen atoms of the nitro group (atom O4) in the 3,5-di­nitro­salicylate anion is disordered over two orientations with occupancy factors of 0.65 (7) and 0.35 (7). Both nitro groups, the phen­oxy­late oxygen atom and a carb­oxy­lic acid group in the anion are coplanar with an r.m.s. deviation of 0.0074 Å. A bifurcated inter­molecular N—H⋯O hydrogen bond [N3—H3*A*⋯O5 = 2.936 (3) Å and N3—H3*A*⋯O6 = 3.153 (3) Å] links the cation and anion in the asymmetric unit.

**Figure 2:**
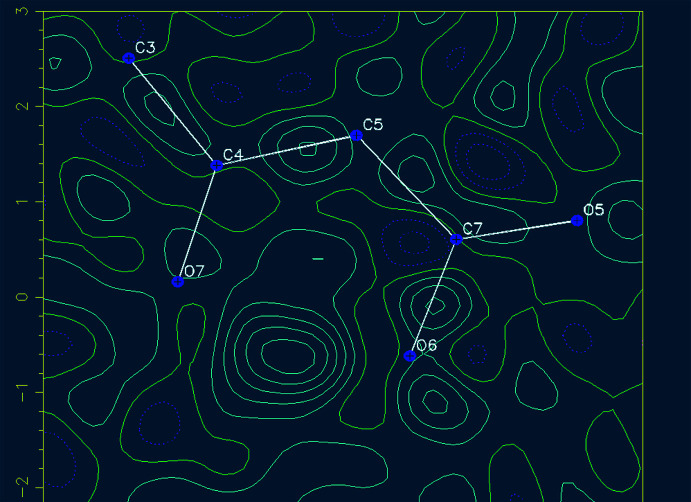
Difference-Fourier electron-density map showing the electron density associated with the H atom involved in the O6—H6⋯O7 hydrogen bond.

In the DNSA mol­ecule, deprotonation of the –COOH group (p*K*_α_ COOH = 2.2) is easier than that of the phenolic –OH group (p*K*_α_ OH = 6.8). 62 carboxyl­ate moiety structures (COO^−^) and 70 phenolate anion structures (O^−^) were found in a search of the Cambridge Structural Database (CSD, Version 5.43, update of March 2020; Groom *et al.*, 2016 ▸), which is perhaps unexpected because the number of crystal structures containing phenolate anions is larger than those containing carboxyl­ate anions. These conflicting results may suggest that the formation of protonated salts of the DNSA mol­ecule with phenolate ions is favoured by the thermodynamic stability and the inter­molecular inter­actions between the phenolate anion and counter-ions in the respective crystal structures. The crystal structure of (I) suggests that the title salt was formed by deprotonation of the phenolic group in the DNSA mol­ecule. In order to better understand the deprotonation of the phenolic group in DNSA mol­ecule, the H-atom electron density in the difference-Fourier electron-density maps was calculated as they can yield additional insight into the proton-transfer behaviour. From Fig. 2 ▸, the electron density associated with atom H6 is shown to be smeared out between the O6 and O7 atoms, with the maximum lying closer to O6 atom than O7. It suggests that the H6*A* atom is attached to the carb­oxy­lic acid group and that deprotonation occurs through the phenolic group. As a result, the strong intra­molecular O6—H6*A*⋯O7 hydrogen bond formed. The inter­atomic distance between the phenolate oxygen atom, O7, and the O6 atom in the carb­oxy­lic acid group is 2.448 (2) Å, which also indicates that the strong intra­molecular hydrogen bond between the O6 and O7 atoms. Similar types of intra­molecular hydrogen bonds were observed in salicylic acid with a distance of 2.62 Å (Woińska *et al.*, 2016 ▸; Montis & Hursthouse *et al.*, 2012 ▸) and in other proton-transfer salts of DNSA in the range 2.409–2.540 Å (Smith *et al.*, 1995 ▸, 1996 ▸, 1997 ▸, 2000 ▸, 2001*a* ▸,*b* ▸,*c* ▸,*d* ▸,*e* ▸, 2002 ▸, 2006 ▸). The proton in the carboxylc acid group is located between the carboxyl-O atom [O6 at 1.14 (3) Å] and the phenolate oxygen atom, [O7 at 1.37 (3) Å]. A similar trend was found in the various proton-transfer salts of DNSA (Smith *et al.*, 2002 ▸).

## 3. Supra­molecular features

**Figure 3:**
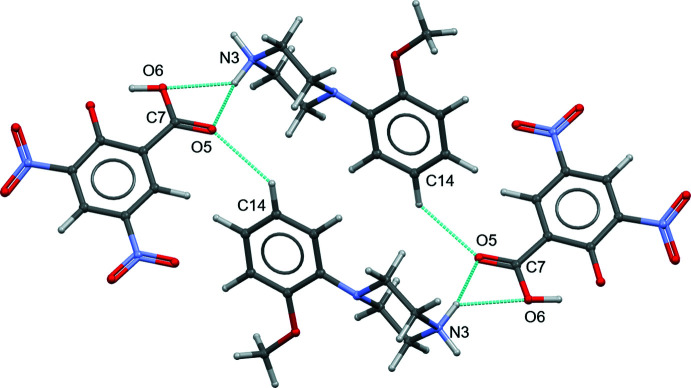
The bifurcated inter­molecular N3—H3*A*⋯(O5,O6) hydrogen bond and the C14—H14⋯O5 inter­action linking the 2MeOPP^+1^ cation and (DNSA)^−1^ anion into a centrosymmetric tetra­mer architecture with an 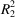 (20) motif.

**Figure 4:**
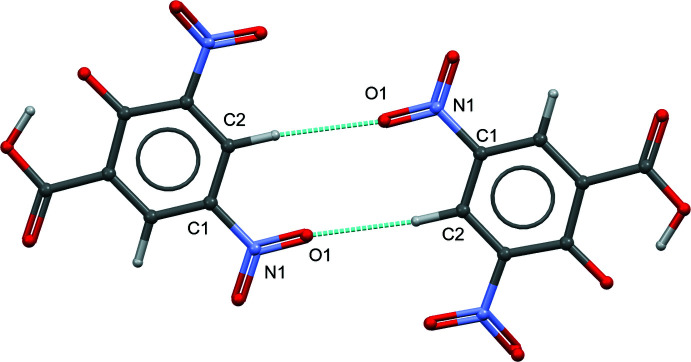
Part of the crystal structure of (I) showing the centrosymmetric dimer motif with the 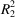 (10) motif formed by the C2—H2⋯O1 inter­action.

**Figure 5:**
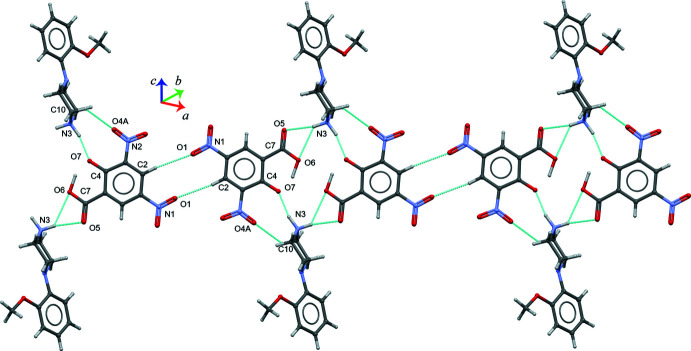
Part of the crystal structure of (I) showing the layered mol­ecular architecture formed by the N3—H3*A*⋯(O5,O6), N3—H3*B*⋯O7 and C10—H10⋯O4*A* inter­actions, which propagates parallel to the *b* axis.

**Figure 6:**
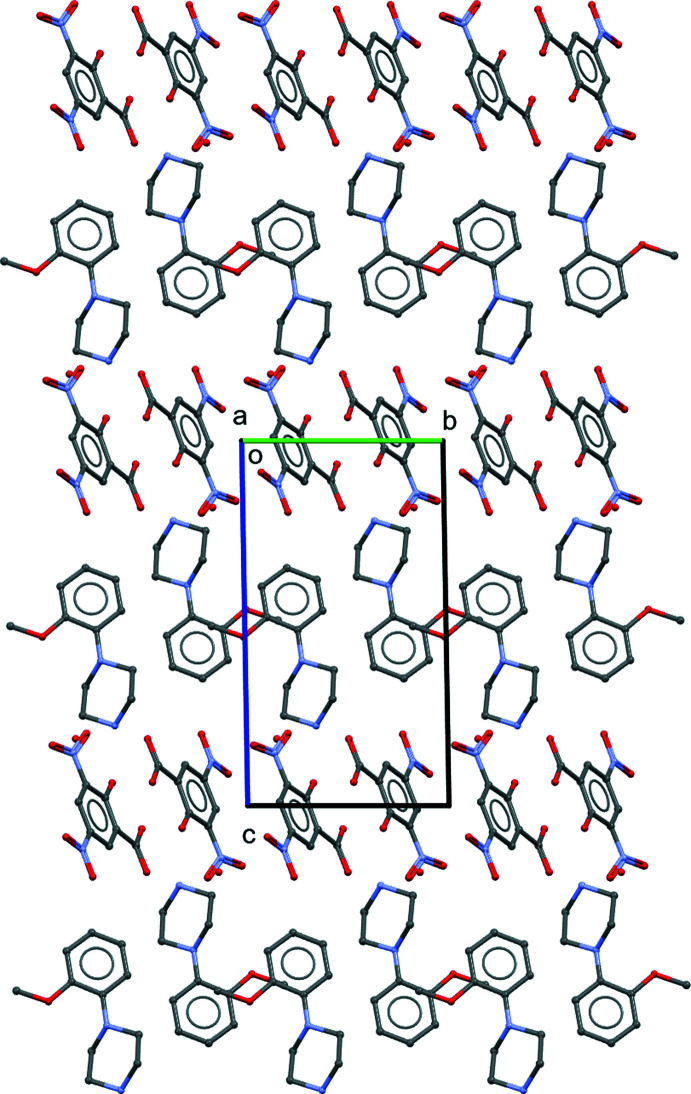
Overall packing diagram for the title salt (I)

The oxygen atoms in both nitro groups (O1–O4), the carb­oxy­lic acid group (O5 and O6) and a phenolate moiety (O7) in the DNSA anion all act as acceptors for various inter­molecular N—H⋯O and C—H⋯O inter­actions, except for atom O4 (Table 1 ▸). In the cation, the O8 atom of the meth­oxy group is not involved in inter­molecular inter­actions. The oxygen atoms of the carb­oxy­lic acid group (O5 and O6) act as acceptors for a bifurcated N3—H3*A*⋯(O5,O6) inter­action, which links two neighbouring cations and anions into a centrosymmetric tetra­meric architecture, which is further stabilized by the C14—H14⋯O5^v^ inter­action [3.481 (3) Å] and yields a macrocyclic ring structure with an 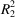 (20) motif (Fig. 3 ▸). Atom O1 of the nitro group is involved in the centrosymmetric C2—H2⋯O1^ii^ inter­action [3.581 (3) Å], which links two neighbouring (DNSA)^1−^ units with an 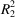 (10) motif (Fig. 4 ▸). Neighbouring dimeric DNSA^1−^ units are further linked through the previously mentioned bifurcated N3—H3*A*⋯(O5,O6) inter­action and the N3—H3*B*⋯O7^i^ [2.787 (3) Å], C10—H10*A*⋯O4*A* [3.118 (10) Å] inter­actions into a layered structure propagating parallel to the *b* axis (Fig. 5 ▸). Of the above three N—H⋯O inter­actions [N3—H3*A*⋯(O5,O6), and N3—H3*B*⋯O7], the N3—H3*B*⋯O7 inter­action is stronger [*D*⋯*A* = 2.787 (3) Å] than the other two, which is due to the fact that two charged components are involved in this inter­action, *i.e.* the phenolate O7 atom in DNSA^−1^ and the protonated N3—H3*B* unit in 2MeOPP^+1^. All of the above inter­actions facilitate the arrangement of the DNSA^1−^ ions in a layered mol­ecular structure. The top and bottom sides of the DNSA^1−^ layers are stabilized by the two adjacent cationic layers. As a result, a sandwich-like arrangement is observed. Briefly, the layered DNSA^1−^ units form the core with the top and bottom sides of the cation layers arranged facing. An overall packing diagram is shown Fig. 6 ▸.

## 4. Hirshfeld surface analysis

**Figure 7:**
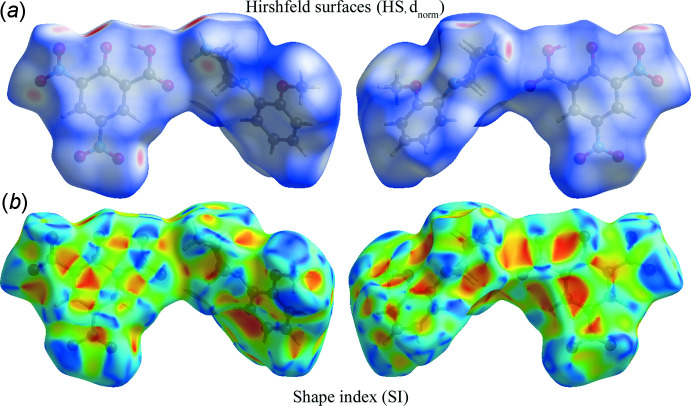
Two different orientations of the Hirshfeld surface of the title salt mapped with (*a*) *d*_norm_ and (*b*) shape index.

**Figure 8:**
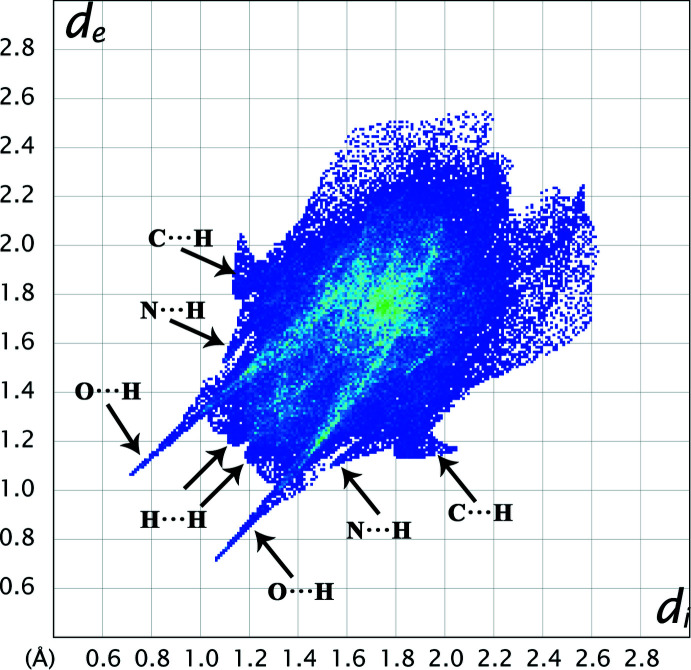
Two-dimensional fingerprint plots for the complete unit of the title salt indicating the various types of contacts.

*Crystal Explorer 17.5* (Turner *et al.*, 2017 ▸) was used to calculate the Hirshfeld surfaces (HS; McKinnon *et al.*, 1998 ▸, 2004 ▸; Spackman & Jayatilaka, 2009 ▸) of the title protonated salt and generate two-dimensional fingerprint plots (full and decomposed, 2D-FP; Spackman & McKinnon, 2002 ▸) in order to investigate and qu­antify the different inter­molecular inter­actions. Distinct colours and intensities indicate short and long contacts, as well as the relative contribution of the different inter­actions in the solid state (Venkatesan *et al.*, 2015 ▸, 2016 ▸). Two views of the HS mapped with *d*_norm_ in the range −0.6295 to 1.3240 a.u. (front and back) are shown in Fig. 7 ▸. Bright red spots on the surface near O2, O3, O4*A*, O7, O6, H10*B* and H3*B* suggest that these atoms participate in hydrogen-bonding inter­actions (see Table 1 ▸). No significant pattern of convex blue and concave red triangles are observed in the shape-index (SI) diagram, indicating the absence of π-stacking inter­actions in the title salt. The 2D-FP plots show the relative contributions of the various non-covalent contacts (Fig. 8 ▸), indicating that inter­molecular O⋯H contacts [sharp symmetrical spikes are observed in the FP plot at *d*_e_ + *d*_i_ = 1.8 Å] make the most significant contribution (38.3%), followed by H⋯H contacts [symmetrical blunt spikes at *d*_e_ + *d*_i_ = 2.4 Å], which contribute 31.8%, while C⋯H, N⋯H, C⋯O, O⋯N, C⋯N and C⋯C contacts contribute 11.6%, 1.7%, 6.7%, 2.7%, 1.9%, 0.5% and 2.8%, respectively. Other significant peaks for various non-covalent contacts are indicated in the FP plot (Fig. 8 ▸).

## 5. Database survey

A search of the Cambridge Structural Database (CSD, Version 5.43, update of March 2020; Groom *et al.*, 2016 ▸) using *Conquest* (Bruno *et al.*, 2002 ▸) for 1-(2-meth­oxy­phen­yl)piperazine gave 111 hits, of which seven hits were for the protonated piperazinium unit. In particular, the crystal structure of 1-(2-meth­oxy­phen­yl) piperazin-4-ium picrate, which like the title compound has a phenolate anion, has been reported (CSD refcode NEBGIK; Verdonk *et al.*, 1997 ▸). In the case of the DNSA mol­ecule, 21 hits were observed for neutral DNSA mol­ecules and 65 and 71 hits for DNSA carboxyl­ate and DNSA phenolate, respectively.

## 6. Synthesis and crystallization

The title protonated salt was synthesized using 1-(2-meth­oxy­phen­yl)piperazine (Sigma Aldrich, 99%) and 3,5-di­nitro­salicylic acid (Merck India, 99.5%) in an equimolar ratio. The stoichiometrically (1 mmol) weighed starting materials were completely dissolved in 50 mL of methanol at room temperature and stirred continuously for 3 h. The homogeneous solution was filtered using Whatmann filter paper and placed in a dust-free atmosphere, and allowed to evaporate slowly at room temperature. A suitable single crystal was harvested after a growth period of 25 days.

## 7. Refinement

Crystal data, data collection and structure refinement details are summarized in Table 2 ▸. The amine H atoms and O-bound H atoms were located in a difference-Fourier map and refined freely along with their isotropic displacement parameters. C-bound H atoms were included in calculated positions and treated as riding atoms [C—H = 0.93–0.98 Å, with *U*_iso_(H) = 1.2*U*_eq_(C)].

## Supplementary Material

Crystal structure: contains datablock(s) I, publication_text. DOI: 10.1107/S2056989022006831/ex2058sup1.cif

CCDC reference: 2183987

Additional supporting information:  crystallographic information; 3D view; checkCIF report

## Figures and Tables

**Table 1:** Hydrogen-bond geometry (Å, °)

*D*—H⋯*A*	*D*—H	H⋯*A*	*D*⋯*A*	*D*—H⋯*A*
O6—H6*A*⋯O7	1.14 (3)	1.37 (3)	2.448 (2)	154 (3)
N3—H3*A*⋯O5	0.94 (4)	2.02 (4)	2.936 (3)	165 (3)
N3—H3*A*⋯O6	0.94 (4)	2.44 (3)	3.153 (3)	133 (2)
N3—H3*B*⋯O7^i^	0.97 (3)	1.83 (3)	2.787 (3)	166 (3)
C2—H2⋯O1^ii^	0.93	2.66	3.581 (3)	174
C9—H9*A*⋯O3^iii^	0.97	2.44	3.254 (4)	141
C10—H10*B*⋯O2^iv^	0.97	2.43	3.319 (3)	152
C10—H10*A*⋯O4*A* ^i^	0.97	2.50	3.118 (10)	122
C14—H14⋯O5^v^	0.93	2.74	3.481 (3)	137
C18—H18*C*⋯N4^vi^	0.96	2.74	3.552 (4)	143

Symmetry codes: (i) 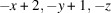 ; (ii) 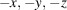 ; (iii) 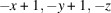 ; (iv) 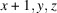 ; (v) 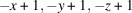 ; (vi) 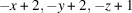 .

**Table 2:** Experimental details

Crystal data	
Chemical formula	C_11_H_17_N_2_O^+^·C_7_H_3_N_2_O^7−^
*M*_r_	420.38
Crystal system, space group	Triclinic, *P* 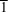
Temperature (K)	296
*a*, *b*, *c* (Å)	7.3729 (6), 8.4842 (7), 15.5411 (13)
α, β, γ (°)	88.954 (4), 81.333 (4), 89.352 (3)
*V* (Å^3^)	960.85 (14)
*Z*	2
Radiation type	Mo *K*α
μ (mm^−1^)	0.12
Crystal size (mm)	0.18 × 0.15 × 0.10

Data collection	
Diffractometer	Bruker Kappa APEXII
Absorption correction	Multi-scan (*SADABS*; Bruker, 2012 ▸)
*T*_min_, *T*_max_	0.608, 0.745
No. of measured, independent and observed [*I* > 2σ(*I*)] reflections	24028, 3513, 2035
*R*_int_	0.084
(sin θ/λ)_max_ (Å^−1^)	0.602

Refinement	
*R*[*F* ^2^ > 2σ(*F* ^2^)], *wR*(*F* ^2^), *S*	0.048, 0.140, 1.02
No. of reflections	3513
No. of parameters	294
No. of restraints	1
H-atom treatment	H atoms treated by a mixture of independent and constrained refinement
Δρ_max_, Δρ_min_ (e Å^−3^)	0.23, −0.21

Computer programs: *APEX2*, *SAINT* and *XPREP* (Bruker, 2012 ▸), *SHELXT2014/5* (Sheldrick, 2015*a* ▸), *SHELXL2018/3* (Sheldrick, 2015*b* ▸), *ORTEP-3 for Windows* (Farrugia, 2012 ▸), *Mercury* (Macrae *et al.*, 2020 ▸) and *PLATON* (Spek, 2020 ▸).

## supplementary crystallographic information

### Crystal data

**Table:**

C_11_H_17_N_2_O^+^·C_7_H_3_N_2_O^7^^−^	*Z* = 2
*M**_r_* = 420.38	*F*(000) = 440
Triclinic, *P*1	*D*_x_ = 1.453 Mg m^−^^3^
*a* = 7.3729 (6) Å	Mo *K*α radiation, λ = 0.71073 Å
*b* = 8.4842 (7) Å	Cell parameters from 3479 reflections
*c* = 15.5411 (13) Å	θ = 2.7–21.4°
α = 88.954 (4)°	µ = 0.12 mm^−^^1^
β = 81.333 (4)°	*T* = 296 K
γ = 89.352 (3)°	BLOCK, yellow
*V* = 960.85 (14) Å^3^	0.18 × 0.15 × 0.10 mm

### Data collection

**Table:**

Bruker Kappa APEXII diffractometer	3513 independent reflections
Radiation source: fine-focus sealed tube	2035 reflections with *I* > 2σ(*I*)
Graphite monochromator	*R*_int_ = 0.084
ω and φ scan	θ_max_ = 25.4°, θ_min_ = 2.4°
Absorption correction: multi-scan (SADABS; Bruker, 2012)	*h* = −8→8
*T*_min_ = 0.608, *T*_max_ = 0.745	*k* = −10→10
24028 measured reflections	*l* = −18→18

### Refinement

**Table:**

Refinement on *F*^2^	Hydrogen site location: mixed
Least-squares matrix: full	H atoms treated by a mixture of independent and constrained refinement
*R*[*F*^2^ > 2σ(*F*^2^)] = 0.048	*w* = 1/[σ^2^(*F*_o_^2^) + (0.0588*P*)^2^ + 0.1844*P*] where *P* = (*F*_o_^2^ + 2*F*_c_^2^)/3
*wR*(*F*^2^) = 0.140	(Δ/σ)_max_ < 0.001
*S* = 1.02	Δρ_max_ = 0.23 e Å^−^^3^
3513 reflections	Δρ_min_ = −0.21 e Å^−^^3^
294 parameters	Extinction correction: SHELXL-2018/3 (Sheldrick 2015b), Fc^*^=kFc[1+0.001xFc^2^λ^3^/sin(2θ)]^-1/4^
1 restraint	Extinction coefficient: 0.050 (4)

### Special details

**Table:**

Geometry. All esds (except the esd in the dihedral angle between two l.s. planes) are estimated using the full covariance matrix. The cell esds are taken into account individually in the estimation of esds in distances, angles and torsion angles; correlations between esds in cell parameters are only used when they are defined by crystal symmetry. An approximate (isotropic) treatment of cell esds is used for estimating esds involving l.s. planes.

### Fractional atomic coordinates and isotropic or equivalent isotropic displacement parameters (Å^2^)

**Table:**

	*x*	*y*	*z*	*U*_iso_*/*U*_eq_	Occ. (<1)
C1	0.2780 (3)	0.2159 (3)	0.07284 (15)	0.0394 (6)	
C2	0.3262 (3)	0.1599 (3)	−0.00995 (15)	0.0403 (6)	
H2	0.245907	0.096812	−0.034491	0.048*	
C3	0.4942 (3)	0.1982 (3)	−0.05608 (14)	0.0381 (6)	
C4	0.6246 (3)	0.2917 (3)	−0.02095 (15)	0.0386 (6)	
C5	0.5670 (3)	0.3451 (3)	0.06598 (14)	0.0357 (6)	
C6	0.3973 (3)	0.3079 (3)	0.11071 (15)	0.0402 (6)	
H6	0.362307	0.344693	0.166821	0.048*	
C7	0.6924 (4)	0.4410 (3)	0.10897 (16)	0.0437 (6)	
C8	0.7613 (3)	0.7961 (3)	0.32628 (14)	0.0453 (7)	
H8A	0.714529	0.899464	0.344577	0.054*	
H8B	0.659791	0.734255	0.312652	0.054*	
C9	0.9053 (4)	0.8122 (3)	0.24697 (16)	0.0547 (8)	
H9A	0.851985	0.862088	0.199882	0.066*	
H9B	1.003753	0.878425	0.260013	0.066*	
C10	1.0516 (4)	0.5698 (3)	0.29246 (16)	0.0528 (7)	
H10A	1.157647	0.624708	0.306580	0.063*	
H10B	1.090079	0.464033	0.274758	0.063*	
C11	0.9068 (4)	0.5609 (3)	0.37158 (15)	0.0449 (7)	
H11A	0.804693	0.498591	0.359076	0.054*	
H11B	0.957081	0.510002	0.419258	0.054*	
C12	0.7467 (3)	0.7377 (3)	0.48169 (14)	0.0349 (6)	
C13	0.6760 (3)	0.6130 (3)	0.53447 (15)	0.0438 (6)	
H13	0.686122	0.511151	0.513072	0.053*	
C14	0.5896 (4)	0.6383 (4)	0.61938 (17)	0.0586 (8)	
H14	0.542766	0.553503	0.654172	0.070*	
C15	0.5738 (4)	0.7860 (4)	0.65120 (17)	0.0644 (9)	
H15	0.514640	0.802436	0.707561	0.077*	
C16	0.6448 (4)	0.9121 (4)	0.60065 (18)	0.0572 (8)	
H16	0.634257	1.013138	0.623208	0.069*	
C17	0.7317 (3)	0.8895 (3)	0.51661 (15)	0.0418 (6)	
C18	0.7951 (4)	1.1646 (3)	0.4922 (2)	0.0782 (10)	
H18A	0.667923	1.193863	0.505074	0.117*	
H18B	0.855921	1.233884	0.447752	0.117*	
H18C	0.851231	1.172510	0.543815	0.117*	
N1	0.0996 (3)	0.1758 (3)	0.12107 (14)	0.0552 (6)	
O4A	0.6481 (12)	0.2063 (16)	−0.1968 (8)	0.079 (3)	0.65
N3	0.9804 (4)	0.6538 (3)	0.21950 (15)	0.0557 (7)	
N4	0.8428 (3)	0.7185 (2)	0.39644 (11)	0.0368 (5)	
O1	−0.0033 (3)	0.0951 (3)	0.08644 (13)	0.0810 (7)	
O2	0.0596 (3)	0.2239 (3)	0.19541 (13)	0.0777 (7)	
O3	0.4386 (3)	0.0337 (3)	−0.16524 (12)	0.0699 (6)	
O5	0.6514 (3)	0.4920 (2)	0.18265 (11)	0.0597 (6)	
O6	0.8538 (2)	0.4713 (2)	0.06426 (11)	0.0584 (6)	
O7	0.7832 (2)	0.3284 (2)	−0.06190 (10)	0.0530 (5)	
O8	0.8105 (2)	1.0068 (2)	0.46251 (12)	0.0556 (5)	
N2	0.5361 (3)	0.1362 (3)	−0.14395 (14)	0.0514 (6)	
O4B	0.690 (2)	0.153 (3)	−0.1854 (16)	0.090 (7)	0.35
H3A	0.891 (5)	0.589 (4)	0.202 (2)	0.087 (11)*	
H3B	1.070 (4)	0.674 (3)	0.168 (2)	0.085 (10)*	
H6A	0.857 (5)	0.415 (4)	−0.002 (2)	0.125 (13)*	

### Atomic displacement parameters (Å^2^)

**Table:**

	*U*^11^	*U*^22^	*U*^33^	*U*^12^	*U*^13^	*U*^23^
C1	0.0373 (14)	0.0422 (15)	0.0384 (14)	−0.0082 (11)	−0.0043 (11)	0.0015 (11)
C2	0.0411 (15)	0.0404 (15)	0.0405 (14)	−0.0013 (12)	−0.0097 (12)	−0.0042 (11)
C3	0.0407 (15)	0.0418 (15)	0.0325 (13)	0.0016 (11)	−0.0071 (11)	−0.0069 (11)
C4	0.0351 (14)	0.0442 (15)	0.0359 (14)	−0.0003 (12)	−0.0030 (11)	−0.0010 (11)
C5	0.0371 (15)	0.0383 (14)	0.0317 (13)	−0.0034 (11)	−0.0044 (11)	−0.0012 (10)
C6	0.0443 (16)	0.0433 (15)	0.0323 (13)	−0.0018 (12)	−0.0035 (11)	−0.0019 (11)
C7	0.0445 (16)	0.0525 (17)	0.0342 (14)	−0.0060 (13)	−0.0057 (12)	−0.0039 (12)
C8	0.0415 (15)	0.0600 (17)	0.0341 (14)	0.0018 (13)	−0.0045 (12)	0.0008 (12)
C9	0.0509 (18)	0.075 (2)	0.0384 (15)	−0.0005 (15)	−0.0074 (13)	0.0067 (13)
C10	0.0530 (18)	0.0567 (18)	0.0472 (16)	0.0080 (14)	−0.0015 (13)	−0.0198 (13)
C11	0.0485 (16)	0.0438 (16)	0.0425 (14)	−0.0002 (12)	−0.0063 (12)	−0.0082 (12)
C12	0.0317 (13)	0.0407 (14)	0.0329 (13)	0.0011 (11)	−0.0064 (10)	−0.0044 (11)
C13	0.0420 (15)	0.0453 (16)	0.0435 (15)	−0.0025 (12)	−0.0048 (12)	0.0021 (12)
C14	0.0474 (17)	0.082 (2)	0.0441 (17)	−0.0007 (15)	−0.0009 (13)	0.0173 (16)
C15	0.059 (2)	0.097 (3)	0.0361 (16)	0.0148 (18)	−0.0058 (14)	−0.0090 (17)
C16	0.0563 (19)	0.063 (2)	0.0547 (18)	0.0131 (15)	−0.0133 (15)	−0.0260 (15)
C17	0.0377 (15)	0.0451 (16)	0.0441 (15)	0.0018 (12)	−0.0097 (12)	−0.0086 (12)
C18	0.069 (2)	0.0371 (18)	0.135 (3)	0.0035 (15)	−0.034 (2)	−0.0234 (18)
N1	0.0508 (15)	0.0625 (16)	0.0497 (14)	−0.0172 (12)	0.0025 (12)	−0.0061 (12)
O4A	0.062 (5)	0.135 (8)	0.039 (2)	−0.029 (4)	0.005 (3)	−0.027 (4)
N3	0.0492 (15)	0.0839 (19)	0.0336 (13)	−0.0120 (14)	−0.0018 (12)	−0.0168 (12)
N4	0.0427 (12)	0.0379 (12)	0.0291 (10)	0.0057 (9)	−0.0038 (9)	−0.0034 (8)
O1	0.0652 (14)	0.1054 (18)	0.0711 (14)	−0.0428 (13)	−0.0005 (11)	−0.0193 (12)
O2	0.0637 (14)	0.1058 (18)	0.0572 (13)	−0.0293 (12)	0.0162 (11)	−0.0244 (12)
O3	0.0679 (14)	0.0792 (15)	0.0646 (13)	−0.0108 (12)	−0.0125 (11)	−0.0313 (11)
O5	0.0585 (12)	0.0813 (14)	0.0388 (10)	−0.0220 (10)	−0.0024 (9)	−0.0154 (9)
O6	0.0467 (12)	0.0858 (15)	0.0409 (11)	−0.0250 (10)	0.0027 (9)	−0.0117 (10)
O7	0.0413 (11)	0.0782 (14)	0.0374 (10)	−0.0139 (9)	0.0025 (8)	−0.0111 (9)
O8	0.0570 (12)	0.0373 (11)	0.0730 (13)	−0.0040 (9)	−0.0105 (10)	−0.0069 (9)
N2	0.0423 (14)	0.0683 (17)	0.0439 (14)	0.0018 (12)	−0.0067 (12)	−0.0136 (12)
O4B	0.048 (7)	0.138 (16)	0.078 (12)	−0.028 (8)	0.023 (7)	−0.052 (9)

### Geometric parameters (Å, º)

**Table:**

C1—C2	1.374 (3)	C11—H11B	0.9700
C1—C6	1.385 (3)	C12—C13	1.384 (3)
C1—N1	1.453 (3)	C12—C17	1.403 (3)
C2—C3	1.374 (3)	C12—N4	1.416 (3)
C2—H2	0.9300	C13—C14	1.395 (3)
C3—C4	1.429 (3)	C13—H13	0.9300
C3—N2	1.460 (3)	C14—C15	1.353 (4)
C4—O7	1.283 (3)	C14—H14	0.9300
C4—C5	1.434 (3)	C15—C16	1.376 (4)
C5—C6	1.373 (3)	C15—H15	0.9300
C5—C7	1.479 (3)	C16—C17	1.381 (3)
C6—H6	0.9300	C16—H16	0.9300
C7—O5	1.225 (3)	C17—O8	1.367 (3)
C7—O6	1.309 (3)	C18—O8	1.422 (3)
C8—N4	1.465 (3)	C18—H18A	0.9600
C8—C9	1.506 (3)	C18—H18B	0.9600
C8—H8A	0.9700	C18—H18C	0.9600
C8—H8B	0.9700	N1—O1	1.217 (3)
C9—N3	1.492 (4)	N1—O2	1.225 (3)
C9—H9A	0.9700	O4A—N2	1.225 (8)
C9—H9B	0.9700	N3—H3A	0.94 (4)
C10—N3	1.487 (3)	N3—H3B	0.97 (3)
C10—C11	1.503 (3)	O3—N2	1.215 (3)
C10—H10A	0.9700	O6—H6A	1.13 (4)
C10—H10B	0.9700	O7—H6A	1.38 (4)
C11—N4	1.452 (3)	N2—O4B	1.228 (13)
C11—H11A	0.9700		
			
C2—C1—C6	121.0 (2)	C13—C12—C17	118.0 (2)
C2—C1—N1	119.0 (2)	C13—C12—N4	123.3 (2)
C6—C1—N1	120.0 (2)	C17—C12—N4	118.6 (2)
C3—C2—C1	119.3 (2)	C12—C13—C14	120.8 (2)
C3—C2—H2	120.4	C12—C13—H13	119.6
C1—C2—H2	120.4	C14—C13—H13	119.6
C2—C3—C4	122.8 (2)	C15—C14—C13	120.1 (3)
C2—C3—N2	116.5 (2)	C15—C14—H14	119.9
C4—C3—N2	120.7 (2)	C13—C14—H14	119.9
O7—C4—C3	124.4 (2)	C14—C15—C16	120.4 (3)
O7—C4—C5	120.4 (2)	C14—C15—H15	119.8
C3—C4—C5	115.3 (2)	C16—C15—H15	119.8
C6—C5—C4	121.2 (2)	C15—C16—C17	120.4 (3)
C6—C5—C7	119.1 (2)	C15—C16—H16	119.8
C4—C5—C7	119.7 (2)	C17—C16—H16	119.8
C5—C6—C1	120.5 (2)	O8—C17—C16	124.5 (2)
C5—C6—H6	119.8	O8—C17—C12	115.3 (2)
C1—C6—H6	119.8	C16—C17—C12	120.2 (2)
O5—C7—O6	120.0 (2)	O8—C18—H18A	109.5
O5—C7—C5	123.1 (2)	O8—C18—H18B	109.5
O6—C7—C5	116.8 (2)	H18A—C18—H18B	109.5
N4—C8—C9	109.1 (2)	O8—C18—H18C	109.5
N4—C8—H8A	109.9	H18A—C18—H18C	109.5
C9—C8—H8A	109.9	H18B—C18—H18C	109.5
N4—C8—H8B	109.9	O1—N1—O2	122.7 (2)
C9—C8—H8B	109.9	O1—N1—C1	118.9 (2)
H8A—C8—H8B	108.3	O2—N1—C1	118.4 (2)
N3—C9—C8	110.1 (2)	C10—N3—C9	110.9 (2)
N3—C9—H9A	109.6	C10—N3—H3A	107 (2)
C8—C9—H9A	109.6	C9—N3—H3A	112 (2)
N3—C9—H9B	109.6	C10—N3—H3B	115.4 (18)
C8—C9—H9B	109.6	C9—N3—H3B	104.9 (17)
H9A—C9—H9B	108.1	H3A—N3—H3B	107 (3)
N3—C10—C11	110.8 (2)	C12—N4—C11	117.54 (18)
N3—C10—H10A	109.5	C12—N4—C8	115.96 (18)
C11—C10—H10A	109.5	C11—N4—C8	110.66 (19)
N3—C10—H10B	109.5	C7—O6—H6A	106.5 (19)
C11—C10—H10B	109.5	C4—O7—H6A	102.6 (15)
H10A—C10—H10B	108.1	C17—O8—C18	118.5 (2)
N4—C11—C10	109.9 (2)	O3—N2—O4A	122.2 (7)
N4—C11—H11A	109.7	O3—N2—O4B	118.7 (15)
C10—C11—H11A	109.7	O3—N2—C3	118.5 (2)
N4—C11—H11B	109.7	O4A—N2—C3	118.2 (8)
C10—C11—H11B	109.7	O4B—N2—C3	119.7 (14)
H11A—C11—H11B	108.2		
			
C6—C1—C2—C3	−1.0 (4)	C15—C16—C17—C12	0.6 (4)
N1—C1—C2—C3	179.6 (2)	C13—C12—C17—O8	177.5 (2)
C1—C2—C3—C4	1.4 (4)	N4—C12—C17—O8	1.3 (3)
C1—C2—C3—N2	−179.4 (2)	C13—C12—C17—C16	−1.4 (4)
C2—C3—C4—O7	179.3 (2)	N4—C12—C17—C16	−177.7 (2)
N2—C3—C4—O7	0.2 (4)	C2—C1—N1—O1	−0.8 (4)
C2—C3—C4—C5	−0.8 (4)	C6—C1—N1—O1	179.8 (3)
N2—C3—C4—C5	−179.9 (2)	C2—C1—N1—O2	178.7 (2)
O7—C4—C5—C6	179.6 (2)	C6—C1—N1—O2	−0.7 (4)
C3—C4—C5—C6	−0.3 (3)	C11—C10—N3—C9	54.4 (3)
O7—C4—C5—C7	−0.9 (4)	C8—C9—N3—C10	−55.3 (3)
C3—C4—C5—C7	179.2 (2)	C13—C12—N4—C11	−14.9 (3)
C4—C5—C6—C1	0.7 (4)	C17—C12—N4—C11	161.1 (2)
C7—C5—C6—C1	−178.8 (2)	C13—C12—N4—C8	119.2 (3)
C2—C1—C6—C5	0.0 (4)	C17—C12—N4—C8	−64.7 (3)
N1—C1—C6—C5	179.3 (2)	C10—C11—N4—C12	−162.6 (2)
C6—C5—C7—O5	−1.3 (4)	C10—C11—N4—C8	61.0 (3)
C4—C5—C7—O5	179.1 (2)	C9—C8—N4—C12	161.0 (2)
C6—C5—C7—O6	178.9 (2)	C9—C8—N4—C11	−61.9 (3)
C4—C5—C7—O6	−0.6 (4)	C16—C17—O8—C18	−3.3 (4)
N4—C8—C9—N3	58.4 (3)	C12—C17—O8—C18	177.8 (2)
N3—C10—C11—N4	−56.8 (3)	C2—C3—N2—O3	−13.1 (3)
C17—C12—C13—C14	1.1 (4)	C4—C3—N2—O3	166.1 (2)
N4—C12—C13—C14	177.1 (2)	C2—C3—N2—O4A	155.4 (6)
C12—C13—C14—C15	0.1 (4)	C4—C3—N2—O4A	−25.4 (7)
C13—C14—C15—C16	−0.9 (4)	C2—C3—N2—O4B	−172.7 (12)
C14—C15—C16—C17	0.5 (4)	C4—C3—N2—O4B	6.4 (13)
C15—C16—C17—O8	−178.2 (2)		

### Hydrogen-bond geometry (Å, º)

**Table:**

*D*—H···*A*	*D*—H	H···*A*	*D*···*A*	*D*—H···*A*
O6—H6*A*···O7	1.14 (3)	1.37 (3)	2.448 (2)	154 (3)
N3—H3*A*···O5	0.94 (4)	2.02 (4)	2.936 (3)	165 (3)
N3—H3*A*···O6	0.94 (4)	2.44 (3)	3.153 (3)	133 (2)
N3—H3*B*···O7^i^	0.97 (3)	1.83 (3)	2.787 (3)	166 (3)
C2—H2···O1^ii^	0.93	2.66	3.581 (3)	174
C9—H9*A*···O3^iii^	0.97	2.44	3.254 (4)	141
C10—H10*B*···O2^iv^	0.97	2.43	3.319 (3)	152
C10—H10*A*···O4*A*^i^	0.97	2.50	3.118 (10)	122
C14—H14···O5^v^	0.93	2.74	3.481 (3)	137
C18—H18*C*···N4^vi^	0.96	2.74	3.552 (4)	143

Symmetry codes: (i) −*x*+2, −*y*+1, −*z*; (ii) −*x*, −*y*, −*z*; (iii) −*x*+1, −*y*+1, −*z*; (iv) *x*+1, *y*, *z*; (v) −*x*+1, −*y*+1, −*z*+1; (vi) −*x*+2, −*y*+2, −*z*+1.
